# Branched-Chain Amino Acid Supplementation Exerts Opposite Effects on Offspring Growth and Differentially Affects Maternal Glucose and Lipid Metabolism in Lactating Dams Fed Normal- or Low-Protein Diets

**DOI:** 10.3390/nu18172867

**Published:** 2026-09-02

**Authors:** Xiuqing Li, Xueyan Lin, Lei Feng, Zhiyong Hu, Qiuling Hou, Yun Wang, Yizhao Shen, Yingyu Mu, Zhonghua Wang

**Affiliations:** Key Laboratory of Efficient Utilization of Non-Grain Feed Resources (Co-Construction by Ministry and Province), Ministry of Agriculture and Rural Affairs, Shandong Provincial Key Laboratory of Animal Nutrition and Efficient Feeding, College of Animal Science and Technology, Shandong Agricultural University, Tai’an 271017, China

**Keywords:** branched-chain amino acids, dietary protein level, lactation, maternal metabolism, offspring growth

## Abstract

**Background**: Branched-chain amino acid (BCAA) is involved in glucose and lipid metabolism and energy homeostasis, but whether its effects on maternal metabolism and offspring growth during lactation depend on dietary protein level remains unclear. **Methods**: C57BL/6J lactating dams were randomized after delivery to a normal-protein control group (CN, 20% protein), low-protein group (LP, 10% protein), CN + BCAA group (CNB), or LP + BCAA group (LPB) (*n* = 11/group). Maternal body composition, glucose and lipid metabolism, hepatic energy status, oxidative stress indices, and pup litter weight gain were assessed. **Results**: The LP diet reduced maternal body weight, pup litter weight gain, and glucose responses during the intraperitoneal pyruvate tolerance test (IPPTT), and increased hepatic lipid deposition and oxidative stress. BCAA supplementation increased pup litter weight gain under normal-protein conditions, but decreased maternal food intake and offspring growth under low-protein conditions. Metabolically, BCAA supplementation reduced the homeostasis model assessment of insulin resistance (HOMA-IR) and hepatic PEPCK1 activity under normal-protein conditions, while partially restoring IPPTT glucose responses and increasing hepatic PEPCK1 activity under low-protein conditions. In addition, BCAA supplementation reduced hepatic lipid deposition and ATP content at both protein levels. Notably, under low-protein conditions, BCAA supplementation increased serum β-hydroxybutyrate (β-HB), while alleviating hepatic oxidative stress. **Conclusions**: BCAA supplementation exerted opposite effects on offspring growth during lactation under different maternal dietary protein levels. These effects may be related to differential changes in maternal glucose and lipid metabolism and hepatic energy status, suggesting that BCAA supplementation during lactation should be evaluated according to maternal dietary protein level.

## 1. Introduction

Lactation is accompanied by substantially increased maternal nutritional demands, and adequate nutrient supply is essential for maintaining maternal metabolic adaptation and offspring growth [1]. Protein serves as a critical substrate for milk synthesis, and lactating mothers require a continuous supply of amino acids and other energy substrates to support milk production and offspring development [2,3,4]. However, inadequate protein intake during lactation continues to be reported in some low- and middle-income regions and among populations with poor dietary quality [5,6]. Therefore, elucidating the effects of insufficient protein intake on maternal metabolic health during lactation and exploring appropriate amino acid-based nutritional intervention strategies hold considerable nutritional significance.

Rodent studies using maternal diets containing 4%, 5%, or 10% protein or casein, relative to respective controls containing 23%, 19%, or 20%, have documented impaired offspring growth together with disturbances in glucose regulation, hepatic lipid handling, and metabolic gene expression [7,8,9]. Beyond its effects on offspring, low-protein intake may also disturb maternal metabolic adaptation during lactation. In Wistar rats fed either a 17% normal-protein diet or a 6% low-protein diet from pregnancy through the end of lactation, the low-protein diet reduced mammary development and prolactin secretion in dams, suggesting that protein insufficiency may impair maternal mammary function during lactation [10]. In Wistar rats, feeding a 10% low-protein diet throughout pregnancy and lactation, with a 20% normal-protein diet serving as the control, resulted in lower maternal body and liver weights, impaired mammary epithelial cell differentiation and liver function, and altered milk composition and lactation performance [11]. More recently, in Sprague Dawley rat dams fed either a 17% normal-protein diet or a 6% low-protein diet throughout pregnancy and lactation, the 6% protein diet induced structural and molecular abnormalities in the liver of dams, accompanied by hepatic stress and disturbed energy metabolism [12]. Together, these findings indicate that low-protein diets can affect both offspring growth and maternal metabolic health during lactation. However, the comprehensive effects of a low-protein diet on glucose and lipid metabolism, hepatic energy status, and oxidative stress in lactating dams remain incompletely understood.

Amino acid supplementation has been explored as a nutritional strategy for modulating metabolic abnormalities associated with protein insufficiency. In pregnant Wistar rats fed either an 18% casein control diet or a 9% casein low-protein diet during pregnancy, supplementation of the low-protein diet with 3% glycine improved low-protein-induced abnormalities in offspring blood pressure [13]. Supplementation with other specific amino acids and amino acid-related compounds, such as taurine, citrulline, alanine or branched-chain amino acid (BCAA), during pregnancy or lactation also affected some aspects of offspring growth, organ development, or metabolic phenotypes [14,15,16,17]. Among these amino acids, BCAA, including leucine, isoleucine, and valine, are essential amino acids and important nutrient-sensing molecules. BCAA function as nutrient signals capable of influencing protein synthesis, carbohydrate and lipid utilization, and cellular energy balance, partly through mTOR-related signaling [18,19]. However, elevated circulating BCAA levels and related metabolites are commonly associated with obesity, insulin resistance, type 2 diabetes, and cardiometabolic diseases [20,21]. In pregnancy-related metabolic disorders, women with gestational diabetes mellitus showed increased serum levels of leucine, isoleucine, and valine in early pregnancy [22]. In contrast, maternal low-protein diets during pregnancy reduced circulating levels of some essential amino acids, particularly BCAA, including leucine, isoleucine, and valine [23]. Under conditions of protein restriction, the physiological effects of BCAA supplementation remain uncertain. Previous work on female ICR mice fed a 20% casein control diet or a 10% casein low-protein diet from 2 weeks before mating throughout pregnancy and lactation showed that BCAA supplementation during pregnancy and lactation under a low-protein diet increased maternal fat mass and hepatic protein content, but did not fully restore low-protein-induced reductions in offspring body and organ weights or alterations in serum biochemical parameters related to protein metabolism [16]. In high-fat-fed obese Sprague Dawley rats receiving a 20% protein control diet or a 5% or 10% low-protein diet during pregnancy and lactation, BCAA supplementation at 100% or 200% of the estimated requirements improved some aspects of maternal energy balance and insulin sensitivity, although it did not restore low-protein-associated offspring growth restriction [17]. More recently, Tzeng et al. reported that a 1.5-fold increase in dietary BCAA content in C57BL/6J dams fed isocaloric, protein content-matched diets containing 20% protein throughout pregnancy and lactation impaired maternal glucose regulation and induced persistent glucose intolerance in adult offspring [24]. These findings suggest that the effects of BCAA supplementation may be context-dependent rather than uniformly beneficial. Whether such context-dependent effects occur during lactation and depend on the underlying dietary protein supply remains unclear.

It remains unclear whether the effects of BCAA supplementation on maternal metabolism and offspring growth during lactation vary with dietary protein level. Accordingly, the present study evaluated the effects of dietary protein level and BCAA supplementation on maternal glucose and lipid metabolism, hepatic energy status, oxidative stress, and offspring growth in lactating dams fed either an AIN-93G-based 20% normal-protein diet or a 10% low-protein diet [25], representing a 50% reduction in dietary protein relative to the normal-protein control. These analyses aimed to clarify the dietary protein-dependent effects of BCAA supplementation during lactation and provide experimental evidence for evaluating the nutritional effects of BCAA supplementation according to maternal dietary protein level.

## 2. Materials and Methods

### 2.1. Animals, Housing, and Diets

The animal study protocol was approved by the Animal Care and Use Committee of Shandong Agricultural University (protocol code SDAUA-2024-236; date of approval: 6 September 2024) and all procedures were conducted in accordance with the ARRIVE 2.0 guidelines.

Specific pathogen-free (SPF) female and male C57BL/6J mice were purchased from Vital River Laboratory Animal Technology Co., Ltd. (Beijing, China) at 8 and 9 weeks of age, respectively. After a one-week acclimation period, the mice were mated at a ratio of three females to one male. Pregnant females were transferred to individual cages and maintained on a standard diet until delivery. The day of delivery was defined as postpartum day 0. Only dams that delivered live pups were included in the lactation experiment. All mice were housed in an animal facility maintained at 25 ± 2 °C under a 12 h light/12 h dark cycle, with free access to food and water. All mice were monitored daily for general health, activity, body condition, and signs of pain or distress.

Sample size was estimated by power analysis based on data from published rodent studies [26,27]. With a two-sided α of 0.05, 90% target power, and equal group allocation, the expected standardized effect sizes for lactational maternal body weight change and offspring body weight at postnatal day 21 were 1.70 and 2.64, corresponding to minimum sample sizes of nine dams per group and five independent litters per group, respectively. The larger estimate was selected, and, with further reference to previous rodent studies involving maternal low-protein diets and BCAA supplementation [9,28], 11 dams were ultimately allocated to each treatment group. Calculations were performed using G*Power version 3.1.9.7.

After delivery, lactating dams were randomly assigned to four treatment groups (*n* = 11/group, total = 44 dams) using a random-number generator: normal-protein control diet (20% protein, CN), low-protein diet (10% protein, LP), normal-protein with BCAA supplementation (CNB), or low-protein with BCAA supplementation (LPB). Based on previous studies [29,30], BCAA supplementation was administered in drinking water at 3% (*w*/*v*). The BCAA mixture consisted of leucine, isoleucine, and valine at a ratio of 2:1:1, corresponding to final concentrations of 1.5%, 0.75%, and 0.75%, respectively. Water intake was recorded throughout lactation. BCAA-containing water intake was recorded daily, whereas plain water intake was recorded every 3 days and converted to average daily intake. Supplemental BCAA intake was calculated based on water consumption and the BCAA concentration [3% (*w*/*v*), 30 mg/mL].

All experimental diets were formulated according to the AIN-93G formulation [25] and were supplied by Jiangsu Xietong Biotechnology Co., Ltd. (Nanjing, China). To generate the low-protein formulation, casein and L-cystine were reduced and the corresponding energy contribution was compensated by increasing corn starch, following the dietary model described previously [16,25]. The normal-protein and low-protein basal diets were isocaloric, with a calculated energy density of 4.0 kcal/g. Their macronutrient energy distributions (protein/carbohydrate/fat) were 20.30%/63.95%/15.75% and 10.15%/74.10%/15.75%, respectively. The CN and CNB groups received the same normal-protein diet, whereas the LP and LPB groups received the same low-protein diet. The complete ingredient composition, macronutrient energy distribution, and calculated energy density of both basal diets are provided in Supplementary Table S1.

### 2.2. Maternal Food Intake, Pup Litter Growth, Body Composition, and Sample Collection

Average daily food intake of lactating dams was calculated as total food intake over the lactation period divided by the number of feeding days and was expressed as g/day/dam. On postpartum day 3, litter size was standardized to seven pups to equalize the lactational demand among dams. Whenever possible, each standardized litter consisted of four male and three female pups to maintain a consistent and balanced sex composition across litters. For litters containing fewer than seven pups, pups were transferred from litters containing more than seven pups within the same treatment group. Any remaining surplus pups were handled in accordance with the approved animal protocol. Cross-fostering was performed only within the same treatment group, and no pups were transferred between different dietary protein or BCAA treatment groups. After standardization, the total litter weight was measured between 10:30 and 12:30 using an electronic balance and was subsequently recorded every 2 days. For each dam, litter growth from postpartum day 3 to 13 was quantified by subtracting the total litter weight recorded on day 3 from that recorded on day 13. A litter was omitted if cannibalism reduced its size to fewer than seven pups during this interval, following an exclusion criterion established before statistical analysis. This exclusion criterion was prespecified before data analysis. On postpartum day 15, after 6 h of fasting, maternal body weight was recorded, and lactating dams were anesthetized with isoflurane (R510-22, RWD Life Science, Shenzhen, China). Maternal body composition was evaluated with an InAlyzer dual-energy X-ray absorptiometry system (Medikors Co., Seongnam, Republic of Korea), and body fat percentage and lean mass were recorded. Retro-orbital blood was subsequently obtained from isoflurane-anesthetized dams and transferred into serum collection tubes containing a clot activator.

After serum separation, samples were visually inspected for hemolysis. Visible hemolysis was identified in three, one, two, and one serum samples from the CN, CNB, LP, and LPB groups, respectively, and these samples were excluded from the corresponding serum biochemical and hormone analyses. When the number of eligible serum samples exceeded that required for a given assay, samples were selected using a random-number generator; otherwise, all eligible samples were included. Different serum aliquots from the same dam could be used for multiple assays; therefore, assay-specific subsets could partially overlap. No sample was selected or excluded according to the magnitude or direction of the measured value, statistical significance, or expected treatment effect.

The dams were then euthanized by cervical dislocation, after which the liver was excised from each dam and weighed. The liver index, defined as liver wet weight relative to maternal body weight, was calculated as follows: liver index (%) = liver wet weight (g)/maternal body weight (g) × 100. Liver samples were stored separately, and samples for hepatic biochemical and RT-qPCR analyses were randomly selected before measurement according to the tissue requirements of each assay.

**Figure 1:**
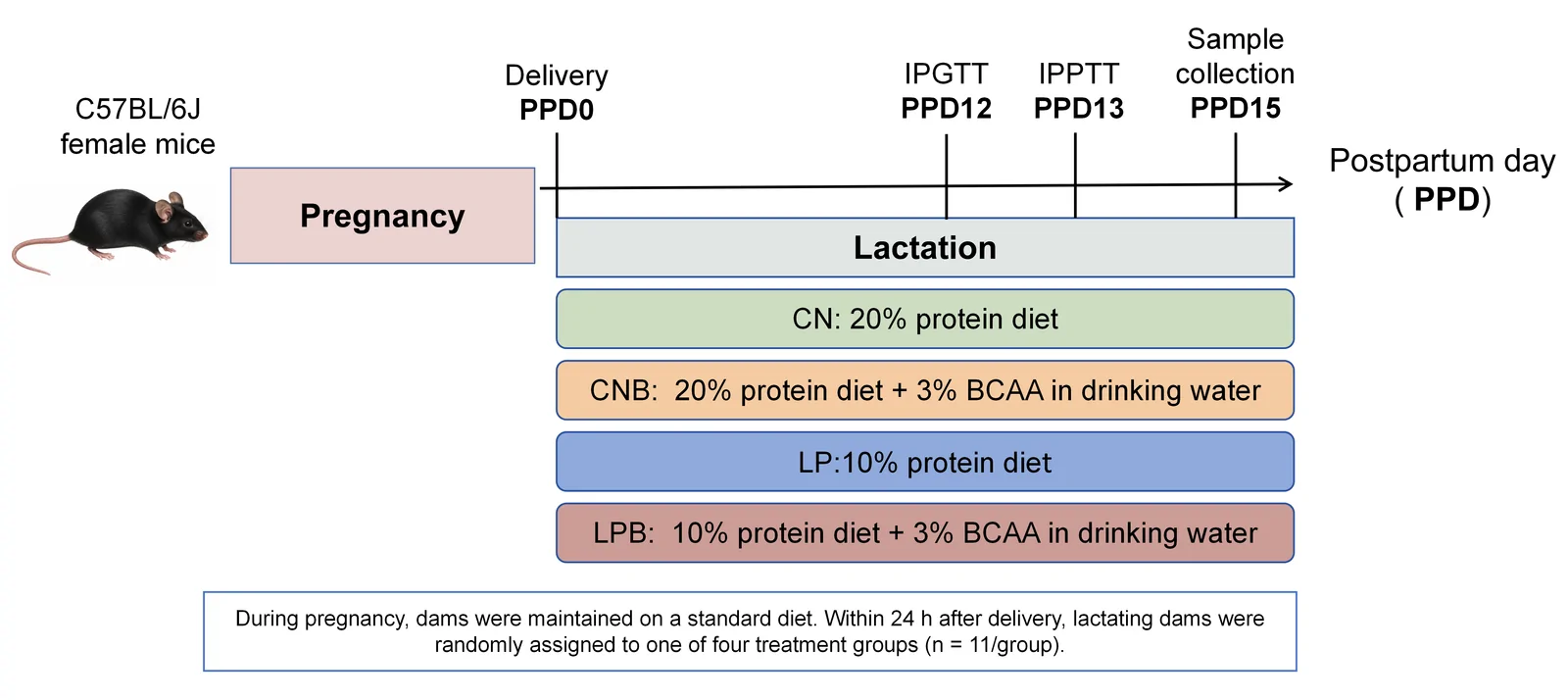
Experimental design.

The overall experimental procedure is illustrated in Figure 1. No blinding to group allocation was performed during the animal intervention.

### 2.3. Intraperitoneal Glucose Tolerance Test (IPGTT) and Intraperitoneal Pyruvate Tolerance Test (IPPTT)

To minimize cumulative stress and potential interference caused by repeated fasting, intraperitoneal injections, and serial tail blood sampling within a short interval, the 11 dams in each treatment group were randomly allocated before testing to two nonoverlapping subsets. Six dams underwent the IPGTT on postpartum day 12, and the remaining five dams underwent the IPPTT on postpartum day 13. This allocation allowed all 11 dams to participate in one of the two metabolic tolerance tests. The IPGTT was performed on postpartum day 12 using D-glucose, whereas the IPPTT was conducted on postpartum day 13 using sodium pyruvate. In both tests, dams were fasted for 12 h and received the corresponding substrate intraperitoneally at 2 g/kg body weight. Tail-blood glucose was measured with a glucometer (Roche Diabetes Care GmbH, Mannheim, Germany ) at 0, 15, 30, 60, 90, and 120 min. AUC was calculated by the trapezoidal method.

### 2.4. Measurement of Serum Biochemical Parameters

Serum biochemical measurements were performed with an automated analyzer (TYPE 7020, Hitachi High-Technologies Corporation, Tokyo, Japan). The measured variables included glucose (GLU), triglyceride (TG), total cholesterol (TC), high-density lipoprotein cholesterol (HDL-C), low-density lipoprotein cholesterol (LDL-C), and albumin (ALB), together with the activities of alanine aminotransferase (ALT), aspartate aminotransferase (AST), and total lactate dehydrogenase (LDH).

### 2.5. Measurement of Metabolic Indices, Hepatic Energy Status, and Oxidative Stress Markers

Serum concentrations of insulin (INS, Cat. No. YJ001983), non-esterified fatty acids (NEFA, Cat. No. YJ025070), and β-hydroxybutyrate (β-HB, Cat. No. YJ251178) were determined using commercial assay kits (Shanghai Enzyme-linked Biotechnology Co., Ltd., Shanghai, China).

For preparation of hepatic homogenates, 100 mg of liver was combined with 0.9 mL phosphate-buffered saline (PBS, pH 7.2–7.4) and homogenized to obtain a 10% (*w*/*v*) suspension. The homogenate was then subjected to centrifugation at 3000 rpm for 20 min at 4 °C in a refrigerated benchtop centrifuge (5427 R, Eppendorf, Hamburg, Germany). After centrifugation, the supernatant was divided into aliquots for the required assays, with unused portions kept at −80 °C until further measurement.

Hepatic adenosine triphosphate (ATP, Cat. No. YJ34490) and adenosine diphosphate (ADP, Cat. No. YJ063251) contents, hepatic phosphoenolpyruvate carboxykinase 1 (PEPCK1, Cat. No. YJ37529) and glucose-6-phosphatase (G6Pase, Cat. No. YJ03761) activities, and serum and hepatic malondialdehyde (MDA, Cat. No. YJ53201) concentrations and superoxide dismutase (SOD, Cat. No. YJ001998) were measured using commercial assay kits. Absorbance was measured at the assay-specific wavelengths using a microplate reader (Tecan, Männedorf, Switzerland). The concentrations or activities of the measured indices were calculated using the corresponding standard curves or calculation procedures provided with the respective kits. The ADP/ATP ratio was calculated from the measured ADP and ATP contents.

Protein concentrations in the liver homogenate supernatants were quantified using a bicinchoninic acid (BCA) assay kit. The working solution was prepared by combining reagents A and B at 50:1 (*v*/*v*). For each measurement, 20 μL of sample was added to 200 μL of the working solution, followed by incubation at 37 °C for 30 min. Absorbance was recorded at 562 nm with a microplate reader, and protein concentrations were derived from a calibration curve prepared with bovine serum albumin. Hepatic assay results were subsequently normalized according to the protein concentration of the corresponding sample.

### 2.6. Reverse Transcription-Quantitative PCR (RT-qPCR)

Total RNA was isolated from liver samples with TRIzol reagent (AG, Changsha, China), and its quantity and purity were evaluated using a NanoDrop spectrophotometer (NanoDrop Technologies, Wilmington, DE, USA). RNA was then reverse-transcribed into cDNA with a commercial kit, followed by SYBR Green-based qPCR (AG, Changsha, China). To ensure reliable normalization, several candidate reference genes were initially evaluated for expression stability across the experimental groups. *β-actin* showed the lowest within-group and between-group variability, with a Ct coefficient of variation below 2% and an amplification efficiency of 99.90%, and was therefore selected as the reference gene. Each biological sample was measured in three or four technical replicate wells. No target-specific amplification was detected in the no-template and no-reverse-transcription controls. Relative target gene expression was calculated using the 2^−ΔΔCt^ method. All primer sequences are listed in Supplementary Table S2 and Supplementary Figure S1.

### 2.7. Calculation of Homeostasis Model Assessment of Insulin Resistance (HOMA-IR)

HOMA-IR was calculated from fasting glucose and insulin concentrations according to the original HOMA model [31] using the following formula:

HOMA-IR = fasting glucose (mmol/L) × fasting insulin (mIU/L)/22.5.

### 2.8. Histological Analysis

For H&E examination, liver specimens were fixed for 24 h in 10% neutral-buffered formalin and processed into 3 μm paraffin sections. Following deparaffinization and rehydration, hematoxylin staining, differentiation, bluing, and eosin counterstaining were conducted according to the specified staining times. Sections were subsequently dehydrated, cleared, mounted, and examined with a bright-field microscope (SWe-CX63; Servicebio, Wuhan, China).

For lipid visualization, 10 μm cryosections were prepared from frozen liver specimens. After fixation, sections were exposed to Oil Red O for 10 min, differentiated in 60% isopropanol, counterstained with hematoxylin, and mounted with glycerol gelatin. Images were obtained under identical microscope settings.

Histology included liver samples from six randomly selected dams per group. For each stain, three non-overlapping microscopic fields per dam were randomly examined at the same magnification. Treatment identities were concealed by coding the slides and images before evaluation; microscopic field selection and quantitative image assessment were subsequently completed without access to group information. Oil Red O-positive areas were quantified using ImageJ software (version 1.54p) and expressed as the percentage of the stained area relative to the total tissue area. Measurements from the three fields were averaged to obtain one value per dam, and the dam was considered the biological replicate.

### 2.9. Statistical Analysis

Data processing, statistical analyses, and graph generation were performed using GraphPad Prism 10.1.2 (GraphPad Software, San Diego, CA, USA) and R 4.5.3 (R Foundation for Statistical Computing, Vienna, Austria). Prior to analysis, normality of model residuals was assessed using the Shapiro–Wilk test and Q-Q plots, and homogeneity of variance was assessed using the Brown–Forsythe test and residuals-versus-fitted plots. When the model assumptions were not satisfied, appropriate data transformations were applied and the assumptions were reassessed. For endpoint variables satisfying the model assumptions, two-way analysis of variance (ANOVA) was performed, with dietary protein level and BCAA supplementation as fixed factors to evaluate their main effects and interaction. When a significant main effect or interaction was detected, pairwise comparisons among the four treatment groups were performed based on estimated marginal means with Tukey adjustment. When residual non-normality persisted after transformation while homogeneity of variance was acceptable, nonparametric tests were applied; when residual normality was acceptable, but heterogeneity of variance remained, generalized least squares (GLS) models were used.

Supplemental BCAA intake was compared between the CNB and LPB groups using a two-sided unpaired Student’s *t*-test.

For IPGTT and IPPTT, AUC was calculated using the trapezoidal method and analyzed according to the same statistical procedure described above for endpoint variables. Blood glucose time-course data from the IPGTT and IPPTT were analyzed using a repeated-measures general linear model, with dietary protein level and BCAA supplementation as between-subject factors and time as the within-subject factor. Greenhouse–Geisser correction was applied when the sphericity assumption was violated. Data are presented as the mean ± standard error of the mean (SEM). *p* < 0.05 was considered statistically significant.

## 3. Results

### 3.1. Effects of Dietary Protein Level and BCAA Supplementation on Maternal Food Intake, Body Composition, and Offspring Growth During Lactation

**Figure 2:**
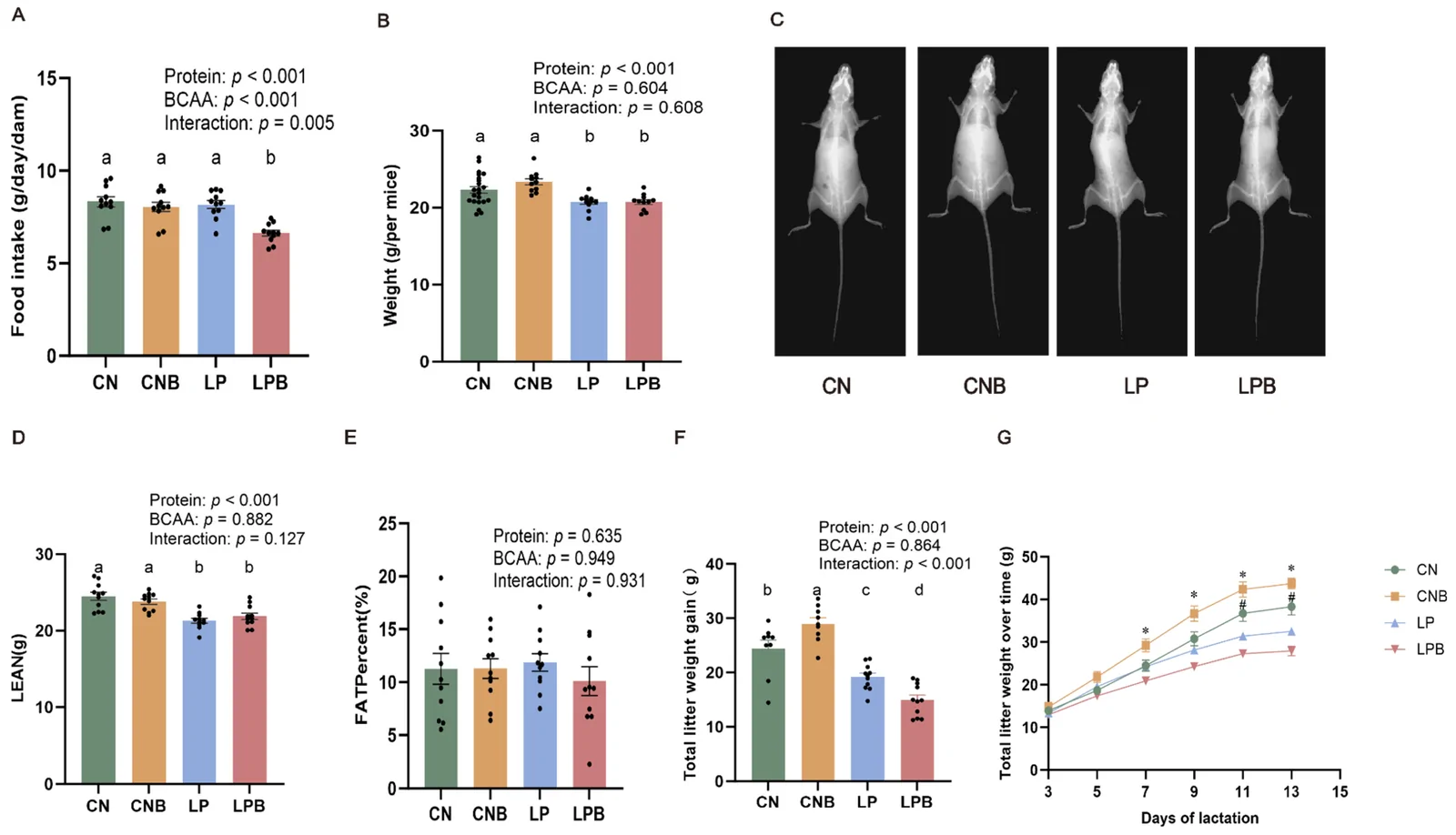
Effects of dietary protein level and branched-chain amino acid (BCAA) supplementation on maternal food intake, body composition, and offspring growth during lactation. (**A**) maternal average food intake; (**B**) maternal body weight at sampling; (**C**) representative images from maternal body composition analysis; (**D**) lean mass; (**E**) body fat percentage; (**F**) total litter weight gain; (**G**) changes in total litter weight over time. Values are expressed as mean ± SEM. Bars not sharing the same letter (a–d) differ significantly at *p* < 0.05. In panel (**G**), * indicates a significant difference between the CN and CNB groups at the same time point (*p* < 0.05), and ^#^ indicates a significant difference between the CN and LP groups at the same time point (*p* < 0.05). *n* = 11 dams per group (**A**–**E**); *n* = 9, 9, 11, and 11 litters for CN, CNB, LP, and LPB, respectively (**F**,**G**).

Maternal food intake, body composition, and offspring growth were evaluated under different dietary protein levels with or without BCAA supplementation. Average maternal food intake was significantly affected by the interaction between dietary protein level and BCAA supplementation (*p* = 0.005; Figure 2A). BCAA supplementation reduced maternal food intake only under low-protein conditions. Water intake did not differ significantly among the four treatment groups, and supplemental BCAA intake from drinking water did not differ significantly between the CNB and LPB groups (*p* ≥ 0.208; Supplementary Figure S2). Maternal body weight and lean mass were affected by dietary protein level alone (*p* < 0.001; Figure 2B,D). Compared with CN dams, LP dams had lower body weight and lean mass, and BCAA supplementation did not attenuate these reductions. Body fat percentage was not significantly affected by dietary protein level, BCAA supplementation, or their interaction (Figure 2E).

Pup litter weight gain was significantly affected by dietary protein level and by the interaction between dietary protein level and BCAA supplementation (*p* < 0.001; Figure 2F). Post hoc comparisons showed that pup litter weight gain was greater in CNB than in CN, whereas it was lower in LPB than in LP. Time course analysis of litter weight showed that CNB litters were heavier than CN litters from postpartum day 7 onward, whereas LP litters were lighter than CN litters from postpartum day 11 onward (Figure 2G). These findings suggest that the effects of BCAA supplementation on maternal food intake and offspring growth depend on dietary protein level.

### 3.2. Effects of Dietary Protein Level and BCAA Supplementation on Glucose Homeostasis

**Figure 3:**
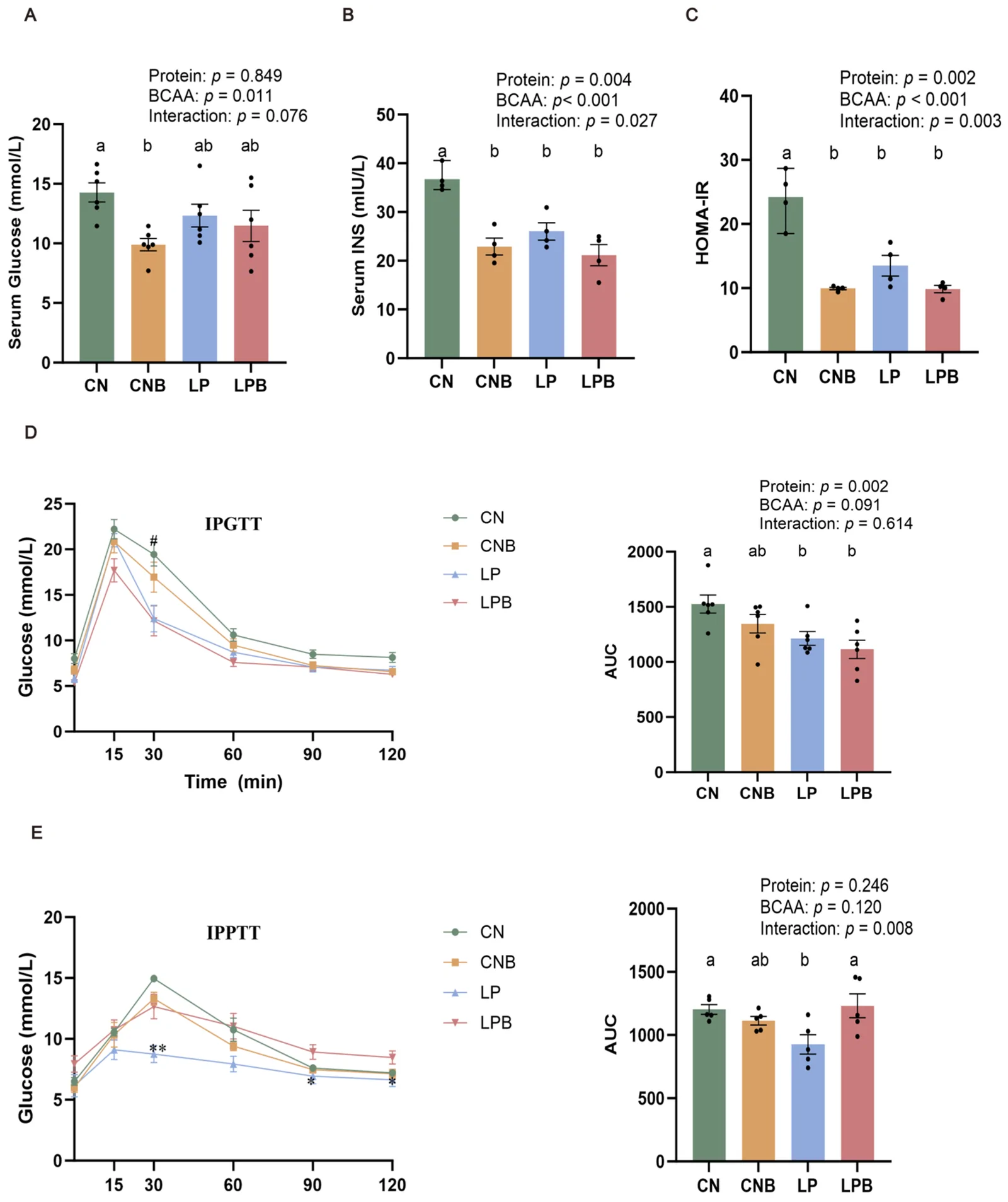
Effects of dietary protein level and BCAA supplementation on glucose homeostasis in lactating dams. (**A**) Fasting serum glucose on postpartum day 15 following a 6 h fast; (**B**) serum insulin concentration; (**C**) homeostasis model assessment of insulin resistance (HOMA-IR) index; (**D**) intraperitoneal glucose tolerance test (IPGTT) curves and corresponding area under the curve (AUC); (**E**) intraperitoneal pyruvate tolerance test (IPPTT) curves and corresponding AUC. Values are expressed as mean ± SEM. Bars not sharing the same letter (a,b) differ significantly at *p* < 0.05. In panel (**D**), ^#^ denotes CN vs. LP (*p* < 0.05). In panel (**E**), ** denotes LP vs. CN, CNB, and LPB (*p* < 0.05), whereas * denotes LP vs. LPB (*p* < 0.05). *n* = 6 dams per group (**A**,**D**); *n* = 4 dams per group (**B**,**C**); and *n* = 5 dams per group (**E**).

Fasting serum glucose showed a significant main effect of BCAA supplementation (*p* = 0.011; Figure 3A). Compared with CN, fasting glucose was significantly lower in CNB. Serum insulin concentration and HOMA-IR showed significant main effects of dietary protein level and BCAA supplementation, as well as a significant interaction between the two factors (*p* ≤ 0.027; Figure 3B,C). Compared with CN, serum insulin concentration and HOMA-IR were significantly lower in CNB, LP, and LPB.

The AUC for the IPGTT was significantly affected by dietary protein level (*p* = 0.002; Figure 3D). Compared with CN, LP showed a significantly lower blood glucose concentration at 30 min and a reduced AUC. In contrast, the glucose response during the IPPTT was significantly affected by the interaction between dietary protein level and BCAA supplementation (*p* = 0.008; Figure 3E). Compared with CN, LP showed significantly lower blood glucose concentrations at 30 min, together with a reduced AUC. Under low-protein conditions, BCAA supplementation increased the blood glucose concentration at 30 min and the AUC. Overall, dietary protein level and BCAA supplementation interacted to affect glucose homeostasis in lactating dams. Notably, BCAA supplementation partially restored the pyruvate-induced glucose response only under low-protein conditions.

### 3.3. Effects of Dietary Protein Level and BCAA Supplementation on Serum Lipid Metabolism

**Figure 4:**
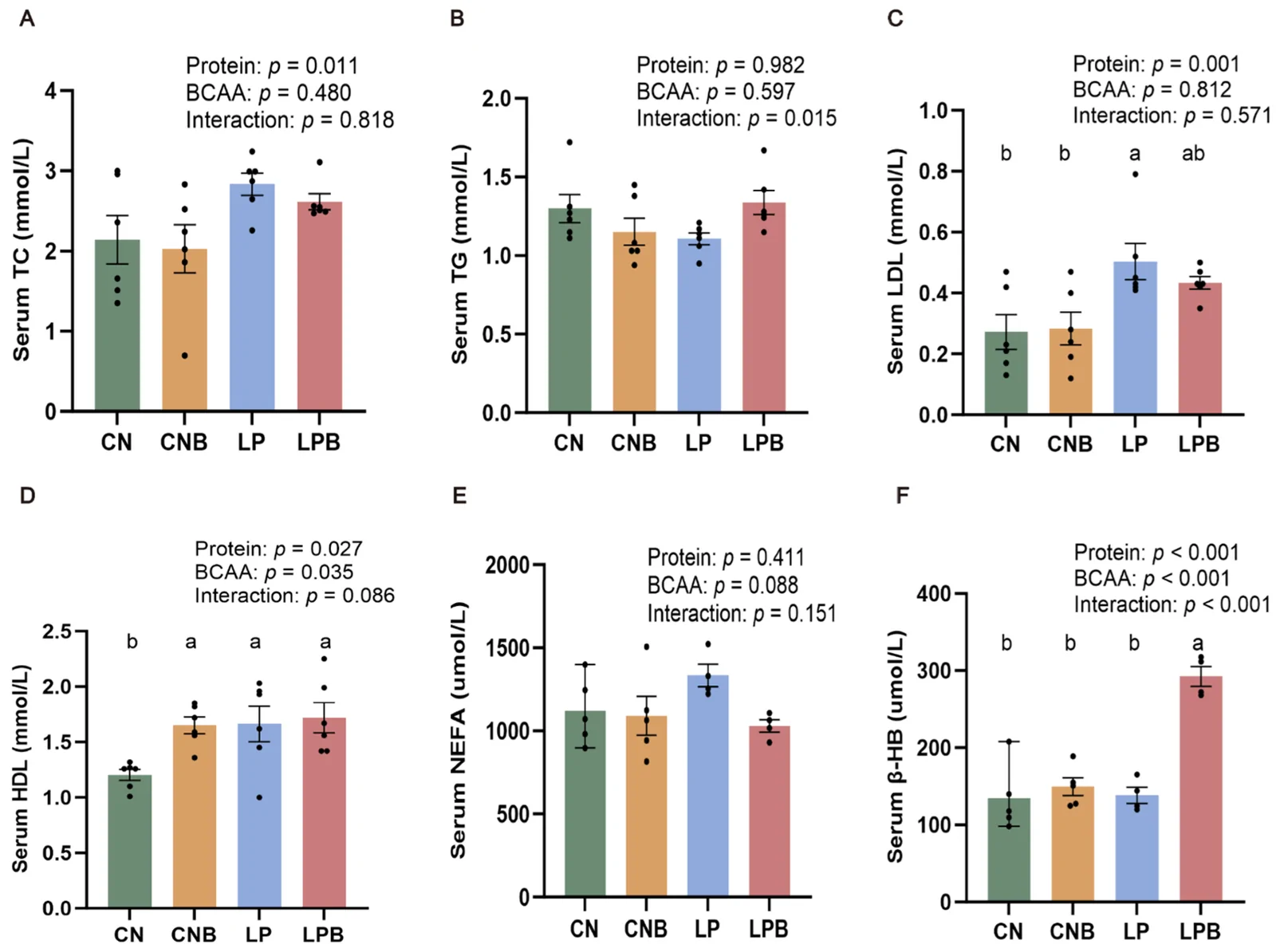
Effects of dietary protein level and BCAA supplementation on serum lipid metabolism in lactating dams. (**A**) Serum total cholesterol (TC) concentration; (**B**) serum triglyceride (TG) concentration; (**C**) serum low-density lipoprotein cholesterol (LDL-C) concentration; (**D**) serum high-density lipoprotein cholesterol (HDL-C) concentration; (**E**) serum non-esterified fatty acid (NEFA) concentration; (**F**) serum β-hydroxybutyrate (β-HB) concentration. Values are expressed as mean ± SEM. Bars not sharing the same letter (a,b) differ significantly at *p* < 0.05. *n* = 6 dams per group (**A**–**D**); *n* = 4 dams per group (**E**,**F**).

Serum TC and LDL-C concentrations showed significant main effects of dietary protein level (*p* ≤ 0.011; Figure 4A,C). Post hoc comparisons showed that LDL-C concentration was significantly higher in LP than in CN. Serum TG concentration was significantly affected by the interaction between dietary protein level and BCAA supplementation (*p* = 0.015; Figure 4B), although post hoc comparisons detected no significant differences among groups. Serum HDL-C concentration showed significant main effects of dietary protein level and BCAA supplementation (*p* ≤ 0.035; Figure 4D). HDL-C concentration was significantly higher in CNB, LP, and LPB than in CN. No significant group differences were detected in serum NEFA concentration, although the main effect of BCAA supplementation showed a trend (*p* = 0.088; Figure 4E). Serum β-HB concentration was significantly affected by dietary protein level, BCAA supplementation, and their interaction (*p* < 0.001; Figure 4F). LPB had significantly higher β-HB concentration than all other groups.

### 3.4. Effects of Dietary Protein Level and BCAA Supplementation on Hepatic Gluconeogenesis and Energy Metabolism

**Figure 5:**
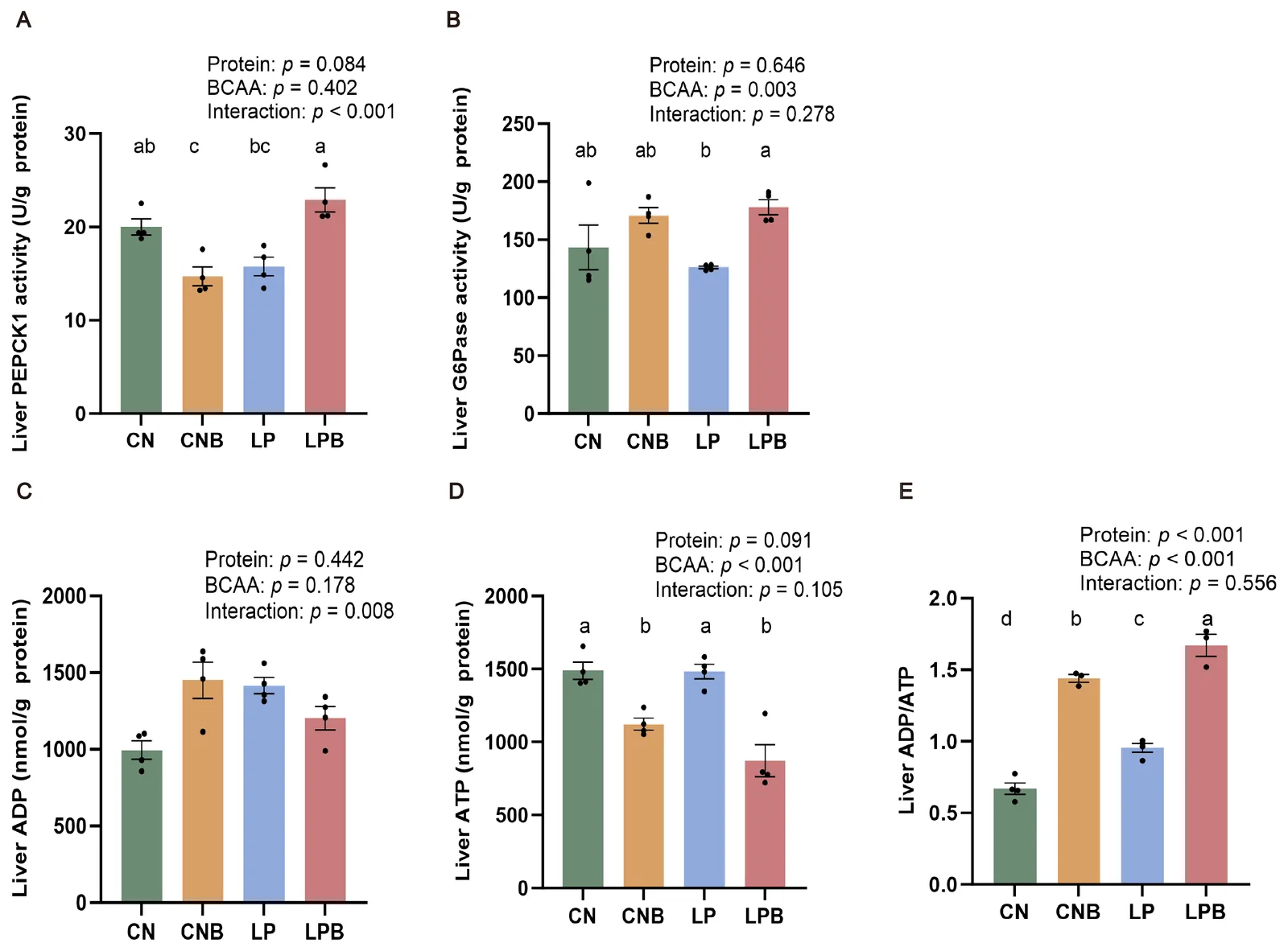
Effects of dietary protein level and BCAA supplementation on hepatic gluconeogenesis and energy metabolism. (**A**) Hepatic phosphoenolpyruvate carboxykinase 1 (PEPCK1) activity; (**B**) hepatic glucose-6-phosphatase (G6Pase) activity; (**C**) hepatic adenosine diphosphate (ADP) content; (**D**) adenosine triphosphate (ATP) content; (**E**) ADP/ATP ratio. Values are expressed as mean ± SEM. Bars not sharing the same letter (a–d) differ significantly at *p* < 0.05. *n* = 4 dams per group (**A**–**D**); *n* = 4, 3, 4, and 3 dams for CN, CNB, LP, and LPB, respectively (**E**).

To assess hepatic gluconeogenesis and energy metabolism, gluconeogenesis-related enzyme activities and hepatic energy status indices were measured. PEPCK1 activity was significantly affected by the interaction between dietary protein level and BCAA supplementation (*p* < 0.001; Figure 5A), whereas G6Pase activity showed a significant main effect of BCAA supplementation (*p* = 0.003; Figure 5B). Post hoc multiple comparisons showed that PEPCK1 activity was significantly lower in CNB than in CN. Under low-protein conditions, BCAA supplementation significantly increased both PEPCK1 and G6Pase activities.

Further analysis of hepatic energy metabolism showed that hepatic ADP content was significantly affected by the interaction between dietary protein level and BCAA supplementation (*p* = 0.008; Figure 5C), although post hoc multiple comparisons detected no significant differences among groups. Hepatic ATP content showed a significant main effect of BCAA supplementation (*p* < 0.001; Figure 5D), while the hepatic ADP/ATP ratio showed significant main effects of dietary protein level and BCAA supplementation (*p* < 0.001; Figure 5E). Compared with CN, the hepatic ADP/ATP ratio was significantly higher in LP. BCAA supplementation significantly reduced hepatic ATP content and increased the hepatic ADP/ATP ratio under both normal-protein and low-protein conditions.

### 3.5. Effects of Dietary Protein Level and BCAA Supplementation on Hepatic Function and Lipid Deposition

**Figure 6:**
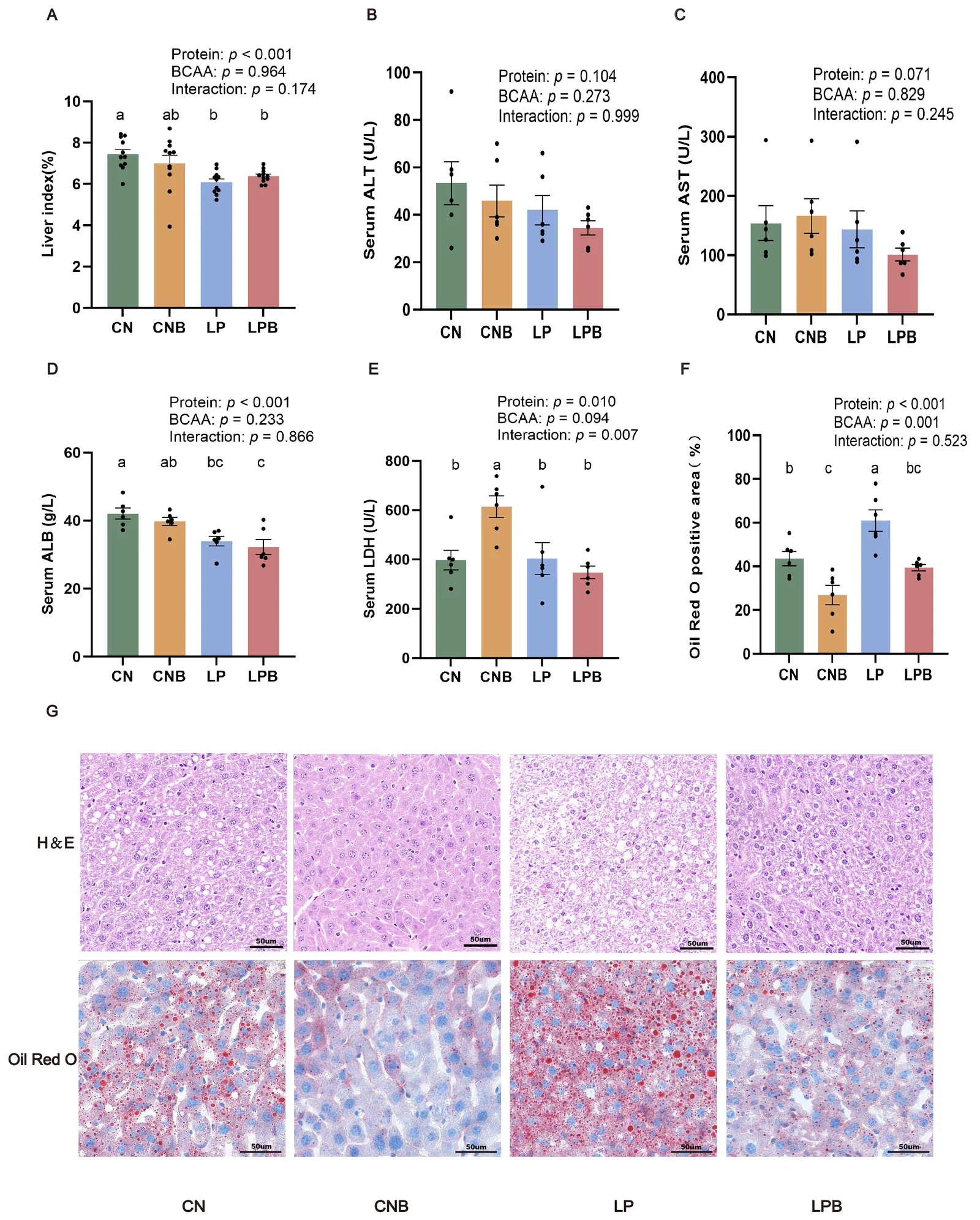
Effects of dietary protein level and BCAA supplementation on liver function and hepatic lipid deposition in lactating dams. (**A**) Liver index; (**B**) serum alanine aminotransferase (ALT) activity; (**C**) serum aspartate aminotransferase (AST) activity; (**D**) serum albumin (ALB) concentration; (**E**) total serum lactate dehydrogenase (LDH) activity; (**F**) quantitative analysis of hepatic Oil Red O-positive area; (**G**) representative images of liver hematoxylin and eosin (H&E) staining and Oil Red O staining. Values are expressed as mean ± SEM. Bars not sharing the same letter (a–c) differ significantly at *p* < 0.05. Scale bars = 50 μm. *n* = 11 dams per group (**A**); *n* = 6 dams per group (**B**–**G**).

Liver index and serum ALB concentration showed significant main effects of dietary protein level (*p* < 0.001; Figure 6A,D). Post hoc comparisons showed that both liver index and serum ALB concentration were significantly lower in LP than in CN. Total serum LDH activity was significantly affected by dietary protein level and by the interaction between dietary protein level and BCAA supplementation (*p* ≤ 0.010; Figure 6E). Compared with CN, total serum LDH activity was significantly higher in CNB. In contrast, serum ALT and AST activities were not significantly affected by dietary protein level, BCAA supplementation, or their interaction (Figure 6B,C).

H&E staining showed that hepatic lobular architecture was relatively intact in CN and CNB, with regularly arranged hepatocytes and only mild vacuolar changes (Figure 6G). In contrast, LP exhibited marked hepatocyte vacuolation accompanied by mild ballooning degeneration. Compared with LP, LPB showed improved hepatic morphology, with reduced hepatocyte vacuolation. Oil Red O staining further showed marked differences in hepatic lipid droplet deposition among groups (Figure 6F,G). Quantitative analysis showed that the Oil Red O-positive area was significantly affected by dietary protein level and BCAA supplementation (*p* ≤ 0.001). Compared with CN, LP showed a significantly larger Oil Red O-positive area. BCAA supplementation significantly reduced the hepatic Oil Red O-positive area under both normal-protein and low-protein conditions.

### 3.6. Effects of Dietary Protein Level and BCAA Supplementation on Serum and Hepatic Oxidative Stress Markers

**Figure 7:**
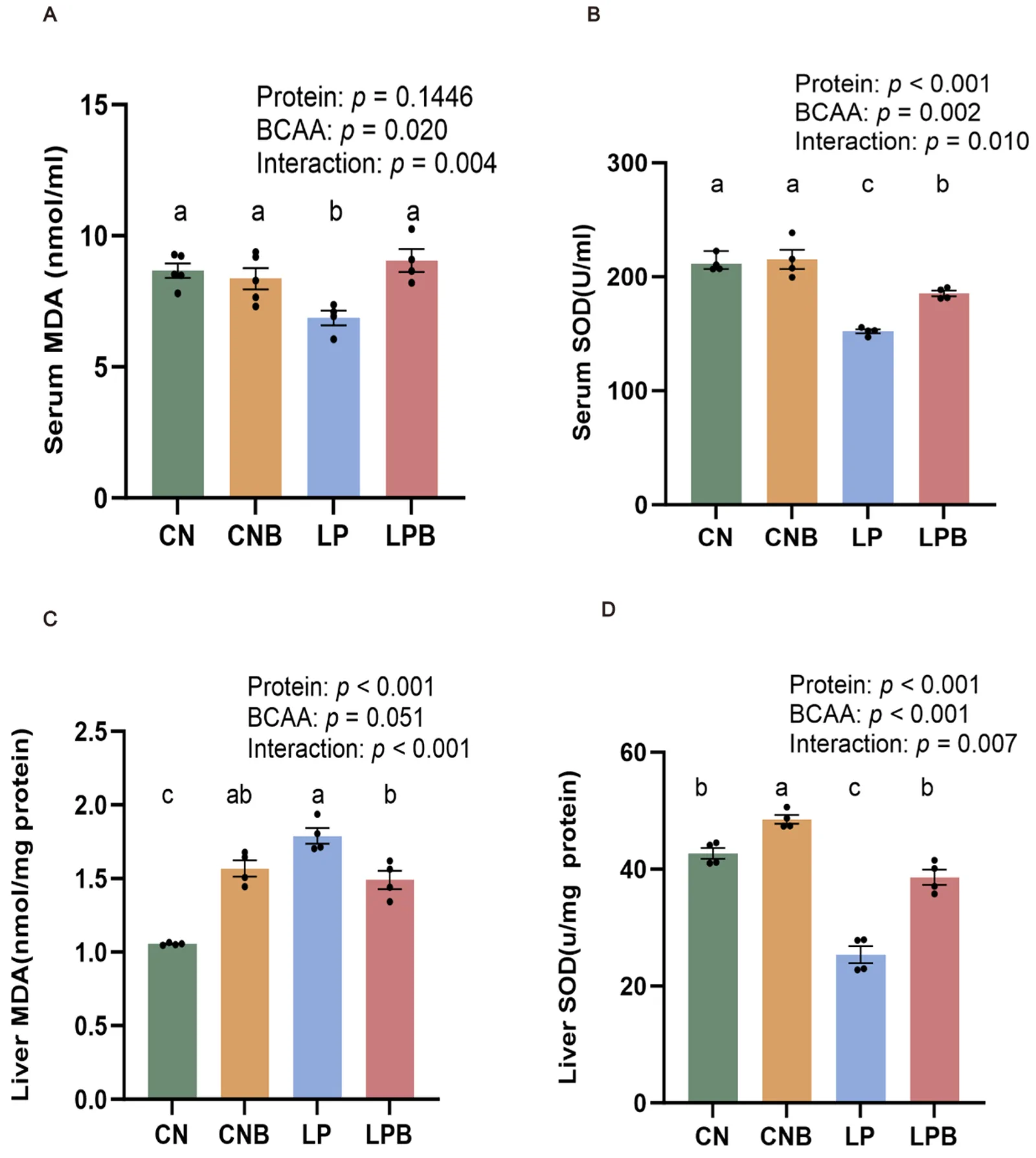
Effects of dietary protein level and BCAA supplementation on serum and hepatic oxidative stress indices in lactating dams. (**A**) Serum malondialdehyde (MDA) concentration; (**B**) serum superoxide dismutase (SOD) activity; (**C**) hepatic MDA content; (**D**) hepatic SOD activity. Values are expressed as mean ± SEM. Bars not sharing the same letter (a–c) differ significantly at *p* < 0.05. *n* = 5 dams per group for CN and CNB and *n* = 4 dams per group for LP and LPB in panel (**A**); *n* = 4 dams per group (**B**–**D**).

Serum MDA concentration showed a significant main effect of BCAA supplementation and a significant interaction between dietary protein level and BCAA supplementation (*p* ≤ 0.020; Figure 7A). Serum SOD activity was significantly affected by dietary protein level, BCAA supplementation, and their interaction (*p* ≤ 0.010; Figure 7B). Post hoc comparisons showed that serum MDA concentration and SOD activity were significantly lower in LP than in CN. Under low-protein conditions, BCAA supplementation significantly increased serum MDA concentration and SOD activity.

In the liver, MDA content was significantly affected by dietary protein level and the interaction between dietary protein level and BCAA supplementation, whereas SOD activity was significantly affected by dietary protein level, BCAA supplementation, and their interaction (*p* ≤ 0.007; Figure 7C,D). Compared with CN, LP showed significantly higher hepatic MDA content and lower SOD activity. Under normal-protein conditions, BCAA supplementation significantly increased hepatic MDA content and SOD activity. Under low-protein conditions, BCAA supplementation significantly reduced hepatic MDA content and increased SOD activity.

### 3.7. Effects of Dietary Protein Level and BCAA Supplementation on the Hepatic mRNA Expression of Antioxidant-Related Genes

**Figure 8:**
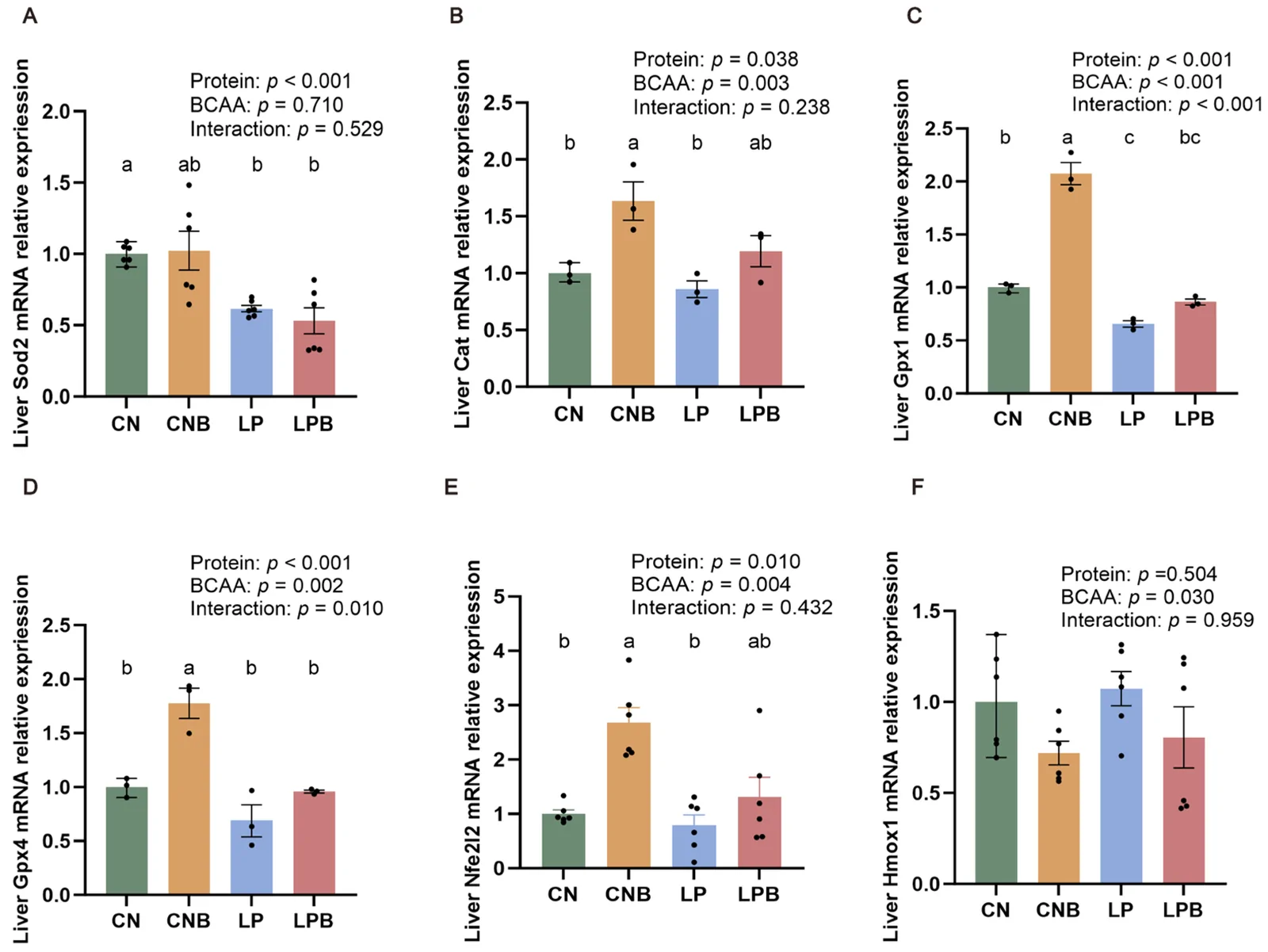
Effects of dietary protein level and BCAA supplementation on the hepatic mRNA expression of antioxidant-related genes in lactating dams. (**A**) Relative hepatic *Sod2* mRNA expression; (**B**) relative hepatic *Cat* mRNA expression; (**C**) relative hepatic *Gpx1* mRNA expression; (**D**) relative hepatic *Gpx4* mRNA expression; (**E**) relative hepatic *Nfe2l2* mRNA expression; (**F**) relative hepatic *Hmox1* mRNA expression. Gene expression was measured by RT-qPCR, normalized to the internal reference gene, and expressed relative to the CN group. Values are expressed as mean ± SEM. Bars not sharing the same letter (a–c) differ significantly at *p* < 0.05. *n* = 6 dams per group (**A**,**E**,**F**); *n* = 3 dams per group (**B**–**D**).

To characterize the transcriptional responses of hepatic antioxidant-related genes to dietary protein level and BCAA supplementation, the relative mRNA expression levels of *Sod2*, *Cat*, *Gpx1*, *Gpx4*, *Nfe2l2*, and *Hmox1* were measured. *Sod2* expression showed a significant main effect of dietary protein level (*p* < 0.001; Figure 8A). *Sod2* expression was significantly lower in LP and LPB than in CN. *Cat* and *Nfe2l2* expression showed significant main effects of dietary protein level and BCAA supplementation (*p* ≤ 0.038; Figure 8B,E). Compared with CN, *Cat* and *Nfe2l2* expression were significantly higher in CNB. Further analysis showed that *Gpx1* and *Gpx4* expression were significantly affected by dietary protein level, BCAA supplementation, and their interaction (*p* ≤ 0.010; Figure 8C,D). Under normal-protein conditions, BCAA supplementation significantly increased *Gpx1*, *Gpx4*, and *Nfe2l2* expression. Under low-protein conditions, BCAA supplementation did not affect the expression of these genes. In addition, *Gpx1* expression was significantly lower in LP than in CN. In contrast, *Hmox1* expression showed only a significant main effect of BCAA supplementation (*p* = 0.030; Figure 8F), although post hoc multiple comparisons detected no significant differences among groups. Overall, BCAA supplementation upregulated the mRNA expression of multiple hepatic antioxidant-related genes under normal-protein conditions, whereas this transcriptional response was attenuated under low-protein conditions.

## 4. Discussion

Maternal protein supply is an established determinant of early offspring growth [8,9,11]. However, how a low-protein diet during lactation affects maternal glucose and lipid metabolism, hepatic energy status, and oxidative stress, and whether BCAA supplementation exerts differential effects under different dietary protein levels, remain insufficiently investigated. The present study showed that the effects of BCAA supplementation on offspring growth and maternal metabolism in dams were clearly dependent on dietary protein level. First, BCAA supplementation increased offspring growth under normal-protein conditions, whereas it further reduced offspring growth under low-protein conditions, accompanied by decreased maternal food intake. Second, the low-protein diet reduced maternal body weight, liver index, and serum ALB concentration, and increased hepatic lipid deposition and oxidative stress. Under normal-protein conditions, BCAA supplementation reduced hepatic lipid deposition and upregulated the mRNA expression of several antioxidant-related genes. Under low-protein conditions, BCAA supplementation also reduced hepatic lipid deposition and partially improved hepatic oxidative status, although it failed to alleviate the decline in maternal body condition. Third, the low-protein diet reduced serum insulin and HOMA-IR and attenuated the glucose responses during the IPGTT and IPPTT. Under normal-protein conditions, BCAA supplementation decreased fasting blood glucose, while under low-protein conditions, it increased gluconeogenesis-related enzyme activities and partially restored the pyruvate-induced glucose response suppressed by the low-protein diet. Overall, under normal-protein conditions, BCAA supplementation increased offspring growth, reduced hepatic lipid deposition, and lowered fasting glucose, serum insulin, and HOMA-IR. Under low-protein conditions, BCAA supplementation partially restored hepatic gluconeogenesis and reduced lipid deposition, but it failed to rescue maternal body condition and was accompanied by reduced food intake and further impaired offspring growth.

Evidence from lactating dams indicates that insufficient maternal protein supply can compromise maternal nutritional status and offspring growth, accompanied by alterations in milk composition, reduced milk production, and poorer litter growth [10,11,32]. In addition, low-protein diets during pregnancy or lactation have been reported to alter maternal hepatic metabolism and reduce final maternal body weight, as well as circulating concentrations of insulin and glucagon [10,11]. Consistent with these findings, the low-protein diet in the present study significantly reduced litter weight gain, maternal body weight, and lean mass, indicating that inadequate protein supply impaired maternal body condition and limited nutritional support for offspring growth during lactation. These results further suggest that the effects of BCAA supplementation on lactation performance depend on the baseline protein supply. Previous studies have reported that BCAA supply is closely associated with mammary amino acid utilization, lactation performance, and offspring growth [16,17,33,34,35]. For example, dietary supplementation with 1.535% or 3.07% BCAA in lactating sows increased milk yield and promoted the growth and survival of suckling piglets [36]. Increasing dietary valine and isoleucine levels also improved weight gain in suckling piglets [37]. Consistent with these reports, BCAA supplementation increased litter weight gain under normal-protein conditions in the present study, suggesting that additional BCAA supply may enhance maternal nutritional support for offspring when basal protein supply is adequate. In contrast, under low-protein conditions, BCAA supplementation from premating through pregnancy and lactation improved some maternal protein metabolism parameters but failed to reverse low-protein-diet-induced growth restriction in offspring. In obese dams, BCAA supplementation during pregnancy and lactation improved some indices of maternal energy balance and insulin sensitivity, but did not rescue impaired offspring growth and development and was accompanied by adverse outcomes, including reduced offspring survival [17]. Consistent with these observations, BCAA supplementation under low-protein conditions during lactation further reduced litter weight gain and was accompanied by lower maternal food intake in the present study. These findings suggest that BCAA supplementation cannot compensate for the lactational limitations caused by insufficient total protein and other essential amino acids.

Previous studies have shown that insufficient dietary protein or amino acid supply can induce disturbances in hepatic lipid metabolism under specific nutritional and physiological conditions. In lactating Sprague Dawley rats, an 8% protein diet induced marked hepatic steatosis and steatohepatitis in dams [38]. Previous research found that providing a diet containing 10% protein throughout gestation and lactation lowered circulating ALB concentrations [16]. Similarly, reducing dietary protein to 5% in male rats resulted in hepatic triglyceride accumulation and lower serum ALB concentrations [39,40,41]. Consistent with these findings, the present study showed that feeding a 10% low-protein diet during lactation reduced serum ALB concentration in dams and aggravated hepatocellular vacuolation and lipid droplet deposition. Meanwhile, serum LDL-C and HDL-C concentrations were increased in the LP group, suggesting that insufficient protein supply affected hepatic lipid deposition and altered the circulating lipid profile. The regulatory role of BCAA supplementation in lipid metabolism has received increasing attention in various nutritional and metabolic models [42,43]. Some studies have shown that BCAA supplementation can alleviate hepatic lipid deposition. For example, BCAA supplementation reduced hepatic steatosis and liver injury in mice with choline-deficient high-fat-diet-induced NASH [44]. Similarly, BCAA supplementation reduced high-fat-diet-induced hepatic lipid accumulation [45]. In contrast, other studies have suggested that excessive or chronically elevated BCAA levels may adversely affect lipid metabolism [42,46]. However, direct comparisons of the effects of BCAA supplementation on maternal hepatic lipid deposition and serum lipid profiles under different dietary protein levels during lactation remain limited. In the present study, BCAA supplementation under normal-protein conditions reduced hepatic lipid deposition and increased litter weight gain, suggesting that it may help maintain hepatic lipid homeostasis and lactational output. Under low-protein conditions, BCAA supplementation also reduced hepatic lipid deposition, but this effect was accompanied by increased serum β-HB concentration, decreased hepatic ATP content, and an elevated ADP/ATP ratio. β-HB is a major ketone body produced through hepatic ketogenesis and is commonly used as an indicator of hepatic fatty acid oxidation [47]. Studies using high-fat diet models have shown that BCAA supplementation can reduce hepatic lipid deposition and upregulate lipid oxidation-related factors, such as PPARα, ATGL, and CPT1A [43,48]. The lower hepatic lipid accumulation observed in LPB dams may therefore partly reflect enhanced fatty acid oxidation and ketone body formation. At the same time, maternal food intake and litter weight gain were both decreased in the LPB group, indicating that the BCAA-induced reduction in hepatic lipid deposition under low-protein conditions may also be influenced by reduced energy intake, altered substrate utilization, and changes in nutrient partitioning, together with metabolic stress.

The effects of low-protein diets on maternal glucose homeostasis have been reported during pregnancy and lactation. Previous studies have shown that low-protein diets during pregnancy [49] or lactation [10] can reduce maternal insulin concentrations. In the present study, serum insulin concentration and HOMA-IR were reduced in the LP group, together with a lower IPGTT AUC, suggesting that a low-protein diet during lactation reduced the post-glucose-load glucose response. However, HOMA-IR is a fasting surrogate index, and HOMA-related indices have limited predictive accuracy for clamp-derived insulin sensitivity in mice [50]. Accordingly, lower fasting glucose or HOMA-IR values alone should not be interpreted as direct evidence of improved insulin sensitivity. Consistent with the reduced post-glucose-load glucose response observed in the LP group, previous studies have likewise reported reduced glucose excursions during glucose tolerance testing in aged mice and in mice fed very-low-protein diets [51,52]. In contrast, another study reported no significant effect of a low-protein diet during pregnancy on the IPGTT glucose response in obese dams [17]. Direct evidence on maternal glucose homeostasis during lactation remains limited, especially with regard to glucose tolerance, pyruvate-induced gluconeogenic response, and hepatic gluconeogenic enzyme activity. Furthermore, the IPPTT glucose response was attenuated in the LP group, suggesting a weakened pyruvate-induced gluconeogenic response. Although hepatic G6Pase activity was not significantly reduced in the LP group compared with the CN group, BCAA supplementation under low-protein conditions increased hepatic PEPCK1 and G6Pase activities and enhanced the IPPTT glucose response. A hepatocyte model showed that amino acid deprivation downregulates G6Pase expression [53], supporting the possibility that amino acid availability can regulate key gluconeogenic enzymes. Consistent with this possibility, studies in fish models suggest that leucine can regulate the expression of gluconeogenesis-related genes. For example, leucine upregulates G6Pase and PEPCK expression in rainbow trout hepatocytes [54], while dietary leucine exerted dose-dependent regulatory effects on hepatic G6Pase and PEPCK expression in juvenile golden pompano [55]. These findings support the possibility that BCAA supplementation may partially restore protein restriction-suppressed gluconeogenesis by regulating key gluconeogenic enzymes. However, the effect of BCAA supplementation on gluconeogenesis appears to be context dependent. In the present study, BCAA supplementation under normal-protein conditions did not enhance the pyruvate-stimulated gluconeogenic response. Further supporting this context-dependent effect, BCAA treatment attenuated the glucose response during the pyruvate tolerance test and suppressed hepatic *Pck1* and *G6pc* mRNA expression in a postsurgical insulin resistance model [56]. Collectively, BCAA supplementation may partially restore hepatic gluconeogenesis under low-protein conditions, whereas this effect was not observed under normal-protein conditions. These findings suggest that the regulation of maternal glucose metabolism by BCAA supplementation during lactation depends on dietary protein level.

A low-protein diet during pregnancy can increase maternal oxidative stress and reduce antioxidant enzyme activity [57]. Pregnancy and lactation protein restriction has also been reported to increase oxidative stress and disrupt antioxidant defense in the offspring’s hypothalamus [58]. A similar pattern was observed in the present study, with LP dams showing greater hepatic MDA accumulation, lower SOD activity, and decreased *Sod2* and *Gpx1* transcript abundance. These findings suggest that a low-protein diet during lactation exacerbated hepatic lipid peroxidation and impaired antioxidant capacity. Notably, serum MDA concentration was reduced in LP, which did not fully align with the hepatic changes. Similar discrepancies between circulating and hepatic oxidative stress markers have also been reported in a chronic stress model, in which serum CAT, SOD, GPx, and MDA levels did not differ significantly, whereas hepatic CAT, SOD, GPx, and MDA levels were increased [59]. A review of oxidative stress markers in NAFLD/NASH also suggests that local hepatic oxidative stress and antioxidant markers do not necessarily change in parallel with blood, plasma, or serum markers [60]. These results indicate that a low-protein diet during lactation altered hepatic oxidative stress status in dams, with divergent changes between serum and hepatic markers. The effects of BCAA supplementation on oxidative stress were strongly dependent on dietary protein level. Under normal-protein conditions, BCAA supplementation did not significantly change serum SOD activity but increased hepatic SOD activity and upregulated the mRNA expression of *Cat*, *Gpx1*, *Gpx4*, and *Nfe2l2*. *Nfe2l2* encodes NRF2, a key transcription factor involved in cellular oxidative stress defense and antioxidant response regulation [61]. These findings are consistent with reports that BCAA or leucine supplementation can alleviate oxidative stress and regulate the expression of NRF2-associated antioxidant enzymes [62,63]. Under low-protein conditions, BCAA supplementation did not significantly restore the expression of most antioxidant-related genes, although hepatic MDA accumulation was lower and SOD activity was higher following BCAA supplementation. This pattern indicates a potential attenuation of hepatic oxidative injury associated with protein restriction.

This study has several limitations. First, the absence of a complete EAA supplemented group precluded direct comparison of BCAA supplementation with a complete EAA mixture, particularly under low-protein conditions. Second, only one dose of BCAA was used within each dietary protein condition, so the optimal supplementation level and dose-dependent effects on oxidative stress and glucose metabolism could not be determined. Third, although serum and hepatic oxidative stress markers and hepatic antioxidant-related gene mRNA expression were measured, the corresponding protein expression levels were not assessed. This limits the extent to which the underlying mechanism can be defined. Fourth, owing to assay-specific sample requirements, biochemical, histological, and gene expression analyses included fewer samples than the initial group size. Samples were randomly selected before analysis, and future studies with larger sample sizes are warranted to further validate these findings. Finally, because this study was conducted in a mouse model, the findings should be extrapolated to clinical settings with caution, as lactating rodents and lactating women differ in protein metabolism, amino acid utilization, and lactation physiology. Future studies should directly compare BCAA supplementation with complete EAA supplementation under matched dietary protein conditions and include graded BCAA doses under different dietary protein levels, tissue-level protein expression and localization analyses, and functional validation, and should be extended to population cohorts or clinical intervention studies. These studies would help clarify how dietary protein level and BCAA supplementation regulate maternal glucose and lipid metabolism, hepatic energy status, and oxidative stress during lactation.

## 5. Conclusions

Overall, this study shows that the response to BCAA supplementation during lactation is shaped by maternal dietary protein level. The low-protein diet impaired maternal body condition, restricted pup litter weight gain, and altered hepatic lipid metabolism, energy status, gluconeogenesis, and oxidative stress. When dietary protein supply was adequate, BCAA supplementation increased pup litter weight gain, reduced hepatic lipid deposition, and upregulated the mRNA expression of several antioxidant-related genes. In contrast, under low-protein conditions, BCAA supplementation partially restored hepatic gluconeogenic activity and reduced hepatic lipid accumulation and oxidative stress, but did not improve maternal body condition. It was also accompanied by lower food intake and a further reduction in offspring growth. These findings indicate that adequate basal protein supply is important for the beneficial effects of BCAA supplementation during lactation. The efficacy and safety of BCAA supplementation under protein-insufficient conditions require further investigation.

## Data Availability

The original contributions presented in this study are included in the article/Supplementary Material. Further inquiries can be directed to the corresponding author.

## Supplementary Materials

The following supporting information can be downloaded at https://www.mdpi.com/article/10.3390/nu18172867/s1, Table S1. Composition of the experimental diets; Table S2. The primer sequences utilized in this study. Figure S1. Melt curves of qPCR amplification for target genes: (A) *β-actin*; (B) *Cat*; (C) *Gpx1*; (D) *Gpx4*; (E) *Hmox1*; (F) *Nfe2l2*; and (G) *Sod2*. Figure S2. Water intake and supplemental BCAA intake in lactating dams: (A) average daily water intake during lactation; (B) average daily supplemental BCAA intake from drinking water in the CNB and LPB groups.

## Abbreviations

The following abbreviations are used in this manuscript:

ADP	Adenosine diphosphate
ALB	Albumin
ALT	Alanine aminotransferase
ANOVA	Analysis of variance
AST	Aspartate aminotransferase
ATP	Adenosine triphosphate
BCAA	Branched-chain amino acid
β-HB	β-Hydroxybutyrate
CN	Normal-protein control diet
CNB	Normal-protein diet plus BCAA
DEXA	Dual-energy X-ray absorptiometry
G6Pase	Glucose-6-phosphatase
H&E	Hematoxylin and eosin
HDL-C	High-density lipoprotein cholesterol
HOMA-IR	Homeostasis model assessment of insulin resistance
IPGTT	Intraperitoneal glucose tolerance test
IPPTT	Intraperitoneal pyruvate tolerance test
LDH	Lactate dehydrogenase
LDL-C	Low-density lipoprotein cholesterol
LP	Low-protein diet
LPB	Low-protein diet plus BCAA
NEFA	Non-esterified fatty acid
PEPCK1	Phosphoenolpyruvate carboxykinase 1
RT-qPCR	Reverse Transcription–Quantitative PCR
SOD	Superoxide dismutase
TC	Total cholesterol
TG	Triglyceride
