# Supplementary material for: Branched-Chain Amino Acid Supplementation Exerts Opposite Effects on Offspring Growth and Differentially Affects Maternal Glucose and Lipid Metabolism in Lactating Dams Fed Normal- or Low-Protein Diets
Source: Nutrients. 2026 Sep 2;18(17):2867. doi: 10.3390/nu18172867 (PMC13567568; doi:10.3390/nu18172867)
Supplement: Supplementary file 1 [file nutrients-18-02867-s001.zip › nutrients-4457856-supplementary.pdf]

Supplementary Table S1. Composition of the experimental diets.

	Diet[1]	
**Composition**	CN	LP
Protein (% Kcal)	20.30%	10.15%
Carbohydrate (% Kcal)	63.95%	74.10%
Fat (% Kcal)	15.75%	15.75%
Calorie density (kcal/g)	4	4
Ingredients (g)		
Casein	200	100
L-Cystine	3	1.5
Corn Starch	397.5	499
Maltodextrin 10	132	132
Sucrose	100	100
Cellulose, BW200	50	50
Soybean oil	70	70
Vitamin Mix V10037	10	10
Mineral Mix S10022G	35	35
Choline Bitartrate	2.5	2.5
Total	1000	1000

[1]The CNB group received the same basal diet as the CN group, and the LPB group received the same basal diet as the LP group. BCAA supplementation was administered in drinking water and was not included in the diet formulation.

Supplementary Table S2. The primer sequences utilized in this study.

Gene[1]	Forward (5′→3′)	Reverse (5′→3′)	Amplification efficiency (%)
β-Actin	GGACTGTTACTGAGCTGCGTT	CGCCTTCACCGTTCCAGTT	99.90
Cat	CCTCGTTCAGGATGTGGTTT	TCTGGTGATATCGTGGGTGA	94.23
Gpx1	GGGACTACACCGAGATGAACGA	ACCATTCACTTCGCACTTCTCA	98.92
GPX4	CGCCAAAGTCCTAGGAAACG	GCACACGAAACCCCTGTACT	103.59
Hmox1	GGTGATGGCTTCCTTGTACC	AGTGAGGCCCATACCAGAAG	104.50
Nfe2l2	TCTTGTCCCGTCCCTAGGTC	AACTTGTACCGCCTCGTCTG	92.56
Sod2	ATTAACGCGCAGATCATGCA	TGTCCCCCACCATTGAACTT	96.62

[1]*β-Actin*, beta-actin; *Cat*, catalase; *Gpx1*, glutathione peroxidase 1; *Gpx4*, glutathione peroxidase 4; *Hmox1*, heme oxygenase 1; *Nfe2l2*, nuclear factor erythroid 2 like 2; *Sod2*, superoxide dismutase 2.

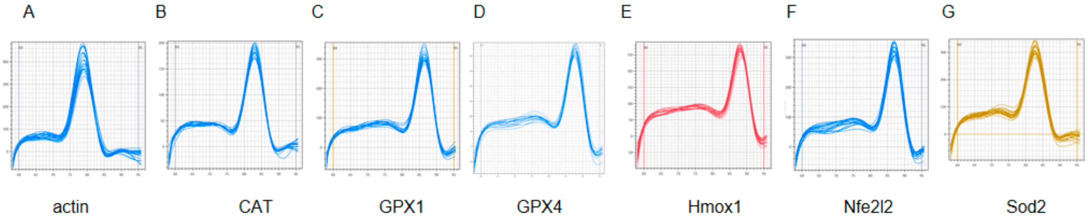

Supplementary Figure S1. Melt curves of qPCR amplification for target genes. (A-G) *β-Actin*, *Cat*, *Gpx1*, *Gpx4*, *Hmox1*, *Nfe2l2*, *Sod2*.

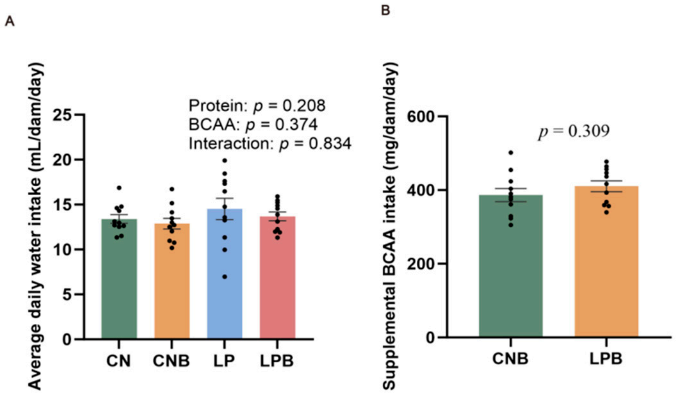

Supplementary Figure S2. Water intake and supplemental BCAA intake in lactating dams. (A) Average daily water intake during lactation. (B) Average daily supplemental BCAA intake from drinking water in the CNB and LPB groups. Supplemental BCAA intake was calculated based on the measured daily water intake and the BCAA concentration in drinking water. Data are presented as the mean ± SEM, n = 11 dams per group. No significant differences were detected ($p > 0.05$).
